# Cutaneous Malignant Melanoma with Testicular Metastases in a Wild Rabbit (*Oryctolagus cuniculus*)

**DOI:** 10.3390/vetsci10070471

**Published:** 2023-07-19

**Authors:** Jessica Maria Abbate, Simone Palazzolo, Antonio Ieni, Giuseppe Santi Rapisarda, Giovanni Lanteri

**Affiliations:** 1 Department of Veterinary Sciences, University of Messina, Polo Universitario Annunziata, 98168 Messina, Italy; jabbate@unime.it; 2 University School for Advanced Studies IUSS Pavia, Piazza della Vittoria, 27100 Pavia, Italy; simone.palazzolo@iusspavia.it; 3 Department of Human Pathology of Adult and Evolutive Age “Gaetano Barresi”, Section of Pathology, University of Messina, 98125 Messina, Italy; 4 Department of Veterinary Prevention, Provincial Health Authority of Catania, 95030 Catania, Italy; grapisarda1975@gmail.com; 5 Department of Chemical, Biological, Pharmaceutical and Environmental Sciences, University of Messina, Polo Universitario Papardo, 98166 Messina, Italy; glanteri@unime.it

**Keywords:** rabbit, *Oryctolagus cuniculus*, malignant melanoma, testicular metastases, HMB-45, Melan-A, S-100

## Abstract

**Simple Summary:**

Oncology in pet rabbits (*Oryctolagus cuniculus*) has emerged in recent years as medical treatments are required with increasing frequency. In this report, we describe a case of cutaneous malignant melanoma occurring in the scrotum in a wild rabbit, with testicular metastases, as an unusual metastatic site only described in human patients to date. Case presentation and gross, histopathological, and immunohistochemical findings are detailed. Descriptions of the biological behaviour of spontaneous tumours may serve to improve current knowledge in animal species and humans in which the same neoplasm occurs. Furthermore, the increasing popularity of rabbits as pets allows for more data on the spontaneous occurrence of cancer.

**Abstract:**

Melanocytic skin tumours have been rarely described in pet rabbits, and exposure to UV light in sparsely haired areas has been hypothesised to play a cancerogenic role. Here, we describe a case of cutaneous malignant melanoma arising from the skin of the scrotum in an 8-year-old male wild rabbit, with testicular metastases as an unusual metastatic site for melanoma reported in humans to date. The tumour was nearly 5 cm in size, firm, and highly pigmented, with multifocal superficial ulcerations and large areas of intratumoural necrosis. The adjacent testis was 1.5 cm, multinodular, and black, obscuring tissue morphology. Histologically, the dermis was expanded by an infiltrative, densely cellular neoplasm composed of nests and sheets of polygonal to spindle neoplastic melanocytes, supported by scant fibrovascular stroma. Neoplastic cells showed intermediate N/C ratio, moderate basophilic cytoplasm, often obscured by abundant brownish granular pigment, and eccentric nuclei with prominent nucleoli. Cellular pleomorphism and nuclear atypia were severe, and high mitotic activity was observed. Diffuse dermal lymphovascular invasion was also observed. The testis was delimited by a thin tunica albuginea, and the parenchyma was largely obscured in its morphology by densely packed neoplastic cells. Seminiferous tubules, lined with a thin basement membrane and containing neoplastic and scattered spermatogenic cells, were occasionally observed. Neoplastic cells within the skin and the testis were positive for HMB-45, Melan-A, and S-100. The growing popularity of rabbits as pets allows for a greater ability to accumulate data on the spontaneous occurrence of tumours in these animals. Furthermore, descriptions of the biological aspects of spontaneously occurring tumours may serve to improve current knowledge in animal species and humans in which the same neoplasm may occur.

## 1. Introduction

Neoplastic disease in pet rabbits (*Oryctolagus cuniculus*) has emerged in recent years as they are allowed to age and medical care is required with increasing frequency [1,2,3]. Indeed, the risk of developing neoplastic disease is positively correlated with aging, and pet rabbits are commonly allowed to live their normal lifespan [1,2,3]. An increasing number of different spontaneous neoplasms have recently been documented in pet rabbits in large-scale retrospective studies, with higher prevalence rates than routinely diagnosed tumours in laboratory rabbits, whose prevalence rates reach 2.7% in animals older than 2 years, as well as in rabbits kept for meat and fur production, otherwise scarified at younger ages [1,2,3,4].

Of note, neoplastic lesions accounted for 81.1% of all surgical biopsies in pet rabbits and 14.4% of all masses examined in autopsied animals in a retrospective analysis of cases, reaching a prevalence rate of up to 47.2% in rabbits older than 6 years [2]. Interestingly, most tumour cases in pet rabbits show histological features of malignancy, with common multiorgan distribution as metastatic or disseminated tumours. Skin cancer is the most common, accounting for 47% of histopathological specimens in pet rabbits, with most cutaneous epithelial tumours reported as benign and most of mesenchymal neoplasms diagnosed as malignant [1,3]. Viral aetiology has been widely documented to be responsible for cases of skin tumours in rabbits, although carcinogenesis is not always associated with viral infection [5]. Both epithelial and mesenchymal skin tumours can be induced by viruses, such as myxosarcoma and Shope fibroma, associated with rabbit fibroma virus infection, also known as Shope fibroma virus, antigenically related to myxoma virus [5,6]. Furthermore, it was postulated that cutaneous mesenchymal tumours could be induced by a vaccine similarly to cats, as aluminium particles were detected within a cutaneous fibrosarcoma [7]. Conversely, it is currently unknown whether the various pet rabbit breeds are prone to a distinct inheritance of susceptibility to neoplastic disease.

Skin tumours are often of epithelial origin, and trichoblastoma is the most frequently diagnosed tumour in rabbits, although mesenchymal tumours, such as soft-tissue sarcomas and fibrosarcomas, have also been frequently observed [1,2,3]. Conversely, cutaneous melanoma is rarely observed in pet rabbits, with a prevalence rate of 4.2% recorded in a retrospective study of 190 cutaneous tumours [3], while it appears to occur more frequently in the oral cavity [8]. Melanocytic tumours in pet rabbits have been observed in the skin of the head, involving the eyelid and ear pinna, and of note, prominent UV exposure in these sparsely haired areas has been postulated to play a cancerogenic role in this animal species as well [1,9]. Melanocytic tumours appear to be a very aggressive type of cancer in this animal species, with lymphovascular invasions recorded in most documented cases and metastases found in regional lymph nodes and lungs or sporadically involving the liver, spleen, and other abdominal organs, leading to melanoma-related death [10,11].

In this case report, we describe a case of cutaneous malignant melanoma arising from the skin of the scrotum with testicular metastases, detailing the case presentation, macroscopic, histopathological, and immunohistochemical features. To the authors’ knowledge, this is the first case of malignant cutaneous melanoma with testicular metastases in a wild rabbit, representing an unusual metastatic site for cutaneous melanoma that has so far been reported in humans as an advanced stage of neoplastic disease.

## 2. Materials and Methods

### 2.1. Case Presentation

An 8-year-old, intact, captive-bred, male, crossbreed, and wild rabbit (*Oryctolagus cuniculus*) weighing 1.8 kg was referred to a private veterinary hospital in the province of Catania (Sicily, Southern Italy) due to a large neoplasm, highly pigmented, and affecting the skin of the scrotum. The mass had occurred several months earlier, and the owners had reported frequent ulcerations and superficial bleeding over the past two months. The animal showed a deterioration in general health over the last 24 h, showing anorexia, lethargy, and apathy.

**Figure 1:**
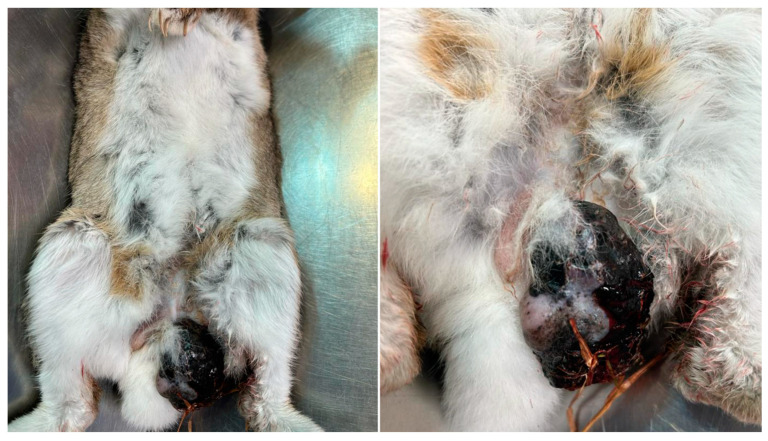
Scrotal melanoma, heavily pigmented, with irregular surface and multifocal ulcerations.

On physical examination, in the inguinal region, affecting the skin of the scrotum, a strongly pigmented mass, approximately 5 cm in diameter, black, with multifocal superficial epidermal ulceration, solid, and firmly adherent to the deep tissues was observed (Figure 1).

Presurgical clinical assessment of the patient’s health status was performed, including a complete physical examination and further diagnostic investigations and laboratory analyses. Specifically, blood was sampled to evaluate the haematological profile and serum biochemistry, and both haematological and biochemical parameters were within the physiological ranges for this animal species (Table 1).

**Figure 2:**
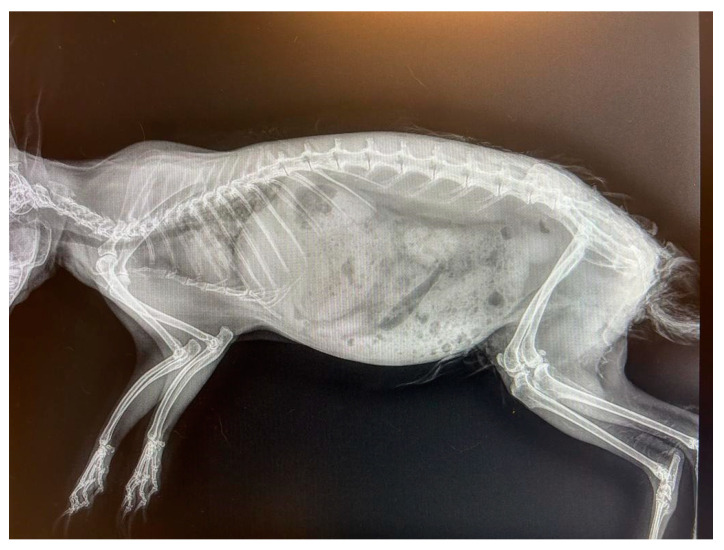
Radiographic examination of the whole body in left lateral projection.

Whole-body radiographic examination, in both left and right lateral views, was performed to exclude the presence of metastases and to estimate the potential success of a surgical excision of the tumour, and no abnormalities were observed (Figure 2).

Furthermore, no other alterations within the abdominal cavity were observed through ultrasonography. In agreement with the owners, aware of factors that may contribute to the rabbit’s overall high susceptibility to anaesthesia-related morbidity and mortality, the patient was admitted for surgery under general anaesthesia. Premedication was performed using meloxicam (0.2 mg/kg, intravenous (IV)); Metacam (Boehringer Ingelheim Animal Health Italia S.p.A); butorphanol (0.2 mg/kg, IV, Dolorex, MSD Animal Health S.r.l.); and medetomidine (0.5 mg/kg, IV, Domitor, Vétoquinol Italia S.r.l.) to provide surgical anaesthesia. An endotracheal tube was placed through an otoscope, and anaesthesia was induced and maintained with isoflurane at 2% (Isoflurane Vet, Boehringer Ingelheim Animal Health Italia S.p.A.). The rabbit was placed in dorsal recumbency, and the surgical area was regularly shaved and disinfected. Skin incisions were made parallel to the tumour, and subcutaneous tissue was bluntly dissected, and the mass was removed with the adjacent, abnormal left testicle. Furthermore, the right testicle was surgical removed, even though it did not show any morphological alterations. Monofilament suture materials (3-0) were used intradermally for closure of subcutaneous and cutaneous layers.

### 2.2. Pathological Examination

The surgically removed scrotal neoplasm and affected left testis were fixed in 10% neutral-buffered formalin and sent to the Department of Veterinary Science of the University of Messina (Italy) for histopathological examination.

After trimming, tissue specimens were routinely processed for histology, embedded in paraffin wax, and 3 μm thick tissue sections were stained with haematoxylin and eosin (HE) for histopathological evaluation.

### 2.3. Immunohistochemistry

Immunohistochemistry (IHC) was performed on 3 μm thick, paraffin-embedded tissue sections, using the Ventana BenchMark ULTRA automated platform with cell conditioning 1 for 64 min and preperoxidase inhibition and primary antibody incubation for 16 min at 37 °C. The OptiView DAB IHC Detection kit (Ventana Medical Systems, Inc., Oro Valley, AZ, USA) was used to detect protein expression of the following primary antibodies: MART-1/Melan A (clone A103, catalogue number 790-2990); anti-Melanosome (clone HMB45, catalogue number 790-4366); and anti-S100 (clone 4C4.9, catalogue number 790-2914). All slides were counterstained with Haematoxylin II (Ventana Medical Systems, Inc.) and Bluing Reagent (Ventana Medical Systems, Inc.) for 4 min at room temperature. Suitable positive controls were used for each IHC reaction, while negative controls were obtained by omitting the specific antisera and substituting PBS for the primary antibody.

## 3. Results

### 3.1. Gross Examination

**Figure 3:**
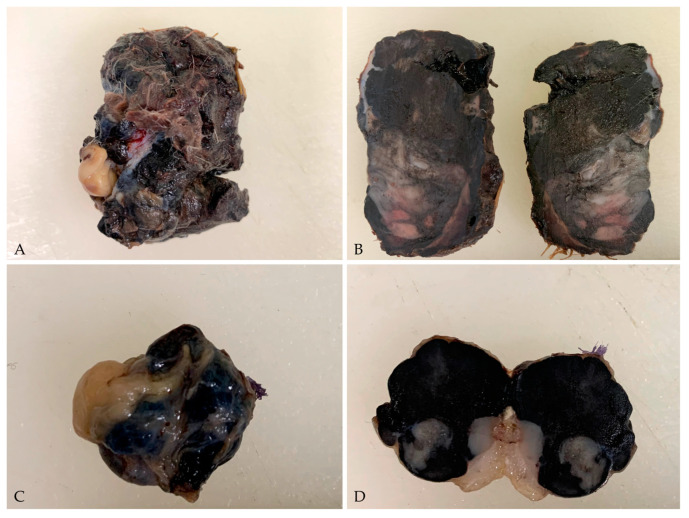
Gross pictures of the cutaneous malignant melanoma and testis. (**A**) The tumour was 5 × 3.5 cm in size, with an irregular cutaneous surface characterised by diffuse hair loss and multifocal ulcerations; (**B**) On the cut surface, the tumour was heavily pigmented with large areas of intratumoural necrosis; (**C**) The testis was 1.5 cm in diameter, multinodular, and intensely black (**D**) On cut surface, the tissue morphology was completely obscured.

On gross examination, the tumour was 5 × 3.5 cm in size, highly pigmented, with an irregular cutaneous surface, characterised by diffuse hair loss and multifocal ulcerations, with a firm and compact texture. On the cut surface, the tumour was black in colour, with large areas of intratumoural necrosis. The testis was 1.5 cm in diameter, multinodular, with high pigmentation on the cut surface that completely obscured tissue morphology (Figure 3). Because the right testis showed no gross changes, it was not referred for histopathological evaluation.

### 3.2. Histopathology

**Figure 4:**
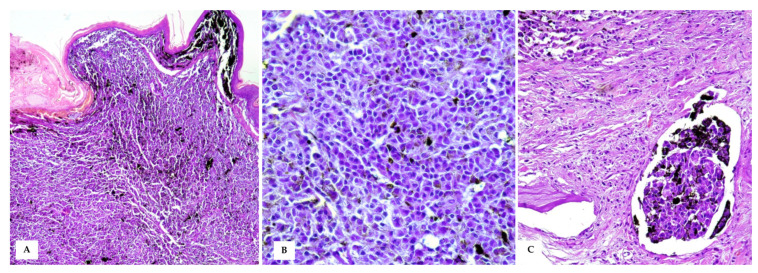
Cutaneous Malignant Melanoma (**A**) A densely cellular neoplasm, markedly expands the dermis and reaches the dermo–epidermal junction, covered by intact or often ulcerated epidermis (HE, 20×); (**B**) At higher magnifications, the neoplastic cells show round to oval nuclei of varying size, moderate amount of cytoplasm with often large amounts of intracytoplasmic melanin, which obscures cellular details (HE, 40×). (**C**) Intratumoural lymphovascular invasion is evident (HE, 40×).

In HE-stained sections, a moderately demarcated, nonencapsulated, infiltrative, densely cellular neoplasm was observed, markedly expanding the dermis and elevating the multifocal ulcerated epidermis, covered by numerous serocellular crusts. The neoplasm was composed of polygonal- to spindle-shaped neoplastic melanocytes arranged in nests and sheets, extending multifocally at the epidermal–dermal junction and supported by scant fibrovascular stroma. Neoplastic cells were 10–15 μm, with often distinct cell borders, intermediate N/C ratio, and moderate, slightly basophilic cytoplasm, often obscured by abundant brownish granular pigment (melanin). Nuclei were round and eccentric, with coarsely stippled chromatin and 1–2 prominent nucleoli. Anisocytosis and anisokaryosis were marked, with karyomegaly, and multinucleated cells were occasionally observed. Mitoses were 18 in 10 high-power fields (400× magnification, 2.37 mm^2^). Diffuse dermal lymphovascular invasion was observed, as well as large areas of intratumoural colliquative necrosis and haemorrhage (Figure 4). Nuclear atypia was >20% (i.e., 33%) [13].

**Figure 5:**
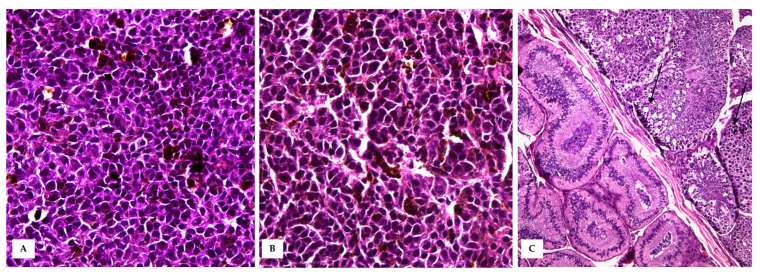
Testicular metastasis. (**A**,**B**) Densely packed polygonal- to spindle-shaped neoplastic melanocytes arranged in sheets, obscuring testicular morphology (HE, 40×). (**C**) Occasionally, markedly expanded seminiferous tubules (black arrows) were observed, lined with a thin basement membrane and filled with polygonal neoplastic cells and degenerated spermatogenic cells (HE, 20×).

The testis was delimited by a thin tunica albuginea, and the testicular parenchyma was largely obscured in its morphology by densely packed neoplastic cells. Occasionally, markedly expanded seminiferous tubules, lined with a thin basement membrane and filled with polygonal neoplastic cells mixed with scattered degenerated spermatogenic cells were observed (Figure 5).

Based on the histopathological findings, a diagnosis of cutaneous malignant melanoma with testicular metastases was made, and the cell immunophenotype was characterised.

### 3.3. Immunohistochemistry

**Figure 6:**
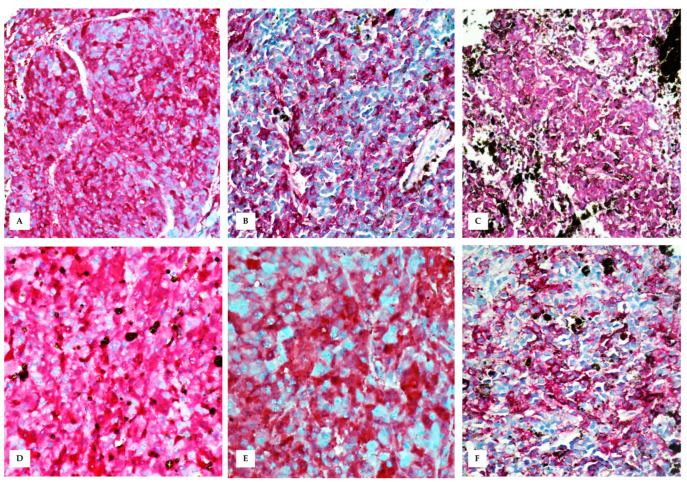
Immunohistochemistry performed on skin tumour (**A**–**C**) and testis (**D**–**F**). (**A**) Immunohistochemical evaluation shows an intense red staining for HMB-45 (nuclear Mayer’s hemalum counterstain, 20×); (**B**) Melan-A (nuclear Mayer’s hemalum counterstain, 20×); (**C**) S-100 (nuclear Mayer’s hemalum counterstain, 20×); (**D**) Intense red staining for HMB-45 in testicular metastasis (nuclear Mayer’s hemalum counterstain, 40×); (**E**) Melan-A (nuclear Mayer’s hemalum counterstain, 40×); and (**F**) S-100 (nuclear Mayer’s hemalum counterstain, 40×).

Neoplastic cells within the skin of the scrotum and within the testis were positive for HMB-45, Melan-A, and S-100 (Figure 6).

## 4. Discussion

In this report, we describe a case of cutaneous malignant melanoma of the scrotum with testicular metastases in a wild rabbit, as an unusual metastatic site only described in human patients to date. The case presentation was detailed, together with pathological and immunohistochemical features. Furthermore, cutaneous melanomas are uncommon neoplasms described in pet and laboratory rabbits, and of note, the New Zealand white rabbit breed appears to be overrepresented [11,14,15]. Recently, in a large retrospective analysis of cases including 330 different neoplasms collected from 290 pet rabbits, 13 were diagnosed as cutaneous melanomas, and the most common anatomical sites of onset included the dorsal trunk, perineal region, and ear pinna [2]. Melanomas were diagnosed in rabbits with a mean age of 62.7 months, and, noteworthy, they were described as aggressively growing tumours with high cellular pleomorphism and mitotic activity [2]. Noteworthy, although few cases of malignant melanoma in rabbits have been reported, lymphovascular invasion, the presence of distant metastases, and tumour recurrence are common pathological findings [11]. Evidence of metastases to multiple organs has been found, including lungs, liver, adventitial surface of the aorta, and submandibular lymph nodes [10,11]. Consequently, death related to malignant melanoma is often reported, thus suggesting that cutaneous melanomas in rabbits are highly malignant [3,11,16].

In veterinary medicine, malignant melanoma commonly occurs in dogs, and the site of occurrence, particularly for the oral cavity, is a crucial determinant of clinical outcome [17]. Additionally, melanocytomas and malignant melanomas account for approximately 12% of scrotal tumours in dogs [18]. Conversely, malignant melanocytic tumours of the skin are rare in other domestic animals, occurring in grey horses and rarely in older cats [17]. Local invasion and metastases to regional lymph nodes and lungs are observed, although spread to unusual sites, such as the spleen, heart, and brain, has occasionally been described [17]. In contrast, in humans, malignant melanoma has the potential to metastasise haematogenously to any organ, and notably, nearly 15% of cutaneous melanomas metastasise in the testis [19]. Notably, although a rare event, testicular metastases should be a primary consideration in patients with a history of cutaneous metastatic melanoma, and the occurrence of cutaneous melanomas as the primary site in testicular metastasis cases ranges from 9% to 41% [19]. Noteworthy, testicular metastases do not appear to be associated with the presence of clinical symptoms in human patients, representing an incidental finding during postmortem investigation [19]. Indeed, malignant melanomas that metastasise to the testis are the most aggressive and life-threatening, representing an advanced stage of neoplastic disease with a generally poor prognosis [20]. Mortality appears extremely high and rapid, with survival of less than 12 months, which explains why most of the data derive from postmortem investigations [19,21].

In veterinary medicine, the testicle represents an uncommon anatomical site for metastatic tumours, and metastatic testicular tumours represent an extremely rare event [17]. Although their rare finding may also be correlated with the widespread neutering of domestic animals at a young age, a real diagnostic underestimation in intact males cannot be excluded because the testicles are not frequently examined organs at autopsy. Lymphoma in dogs, horses, and bulls and hemangiosarcoma in dogs and wild boars have been described [17]. In contrast, in human patients, primary tumours that metastasise to the testicles are common, most frequently including prostate adenocarcinomas (30–65%), followed by pulmonary carcinomas (20%), cutaneous melanomas (10%), and, rarely, renal, upper respiratory, and gastrointestinal carcinomas (<10%) [21]. Metastatic tumours spread to the testis through direct contact or by retrograde venous and lymphatic routes and by arterial embolism. Histopathological findings that may raise suspicion of testicular metastases include (1) lymphovascular neoplastic emboli within the testis, epididymis, and spermatic cord; (2) neoplastic cell morphology not indicative of primary testicular tumours; and (3) misleading findings, such as predominant intratubular extension mimicking intratubular germ cell neoplasia invading the rete testis, occurring in 27% of human cases [22].

In pet rabbits, primary testicular neoplasms are rarely reported, mainly because males are usually neutered in the first few years of life to prevent reproduction and decrease social aggression [23]. Among testicular tumours in rabbits, granular cell tumours are the most-represented, followed by seminoma and interstitial cell tumours [2,23,24]. In addition, testicular gonadoblastoma, Sertoli cell tumour, and teratoma have been occasionally reported in pet rabbits [2,25,26]. In the case described here, the testis was macroscopically altered in its morphology and intensely pigmented. Histologically, the testicular parenchyma was obscured by densely packed neoplastic cells with generally diffuse and only occasional intratubular distribution. The neoplastic cells often contained black to brownish granular cytoplasmic pigment, allowing any differential diagnosis to be ruled out. In addition, neoplastic cells in the skin of the scrotum and within the testis were positive for three melanocyte markers: HMB-45, Melan-A (MART-1), and S-100. There are only few reports concerning the immunophenotype of rabbit neoplasms; however, the utility of human markers for characterizing the immunophenotype of melanocytes in pet rabbits has been previously demonstrated [10], and because none of these markers have 100% specificity, higher diagnostic accuracy would require the use of multiple markers to improve diagnostic accuracy.

Conservative surgery remains the treatment of choice for rabbit cancer if the tumour has not spread to other parts of the body, although anaesthesia-related surgical complications are commonly reported in pet rabbits as well as postoperative complications related to stress and/or pain [27,28]. Chemotherapy protocols have shown variable success when used in pet rabbits, and several side effects have been reported from various chemotherapeutics administered to pet rabbits, including gastrointestinal stasis, anaemia, inappetence, and manifestation of subclinical pasteurellosis or encephalitozoonosis [29]. Although the efficacy of several innovative therapeutic approaches has been tested primarily in laboratory rabbits as animal models of different types of tumours, most veterinarians do not recommend chemotherapy as a treatment option for tumours in pet rabbits [30,31]. Therefore, monitoring a rabbit’s health remains the best way to prevent cancer.

## 5. Conclusions

Here, we describe a case of cutaneous malignant melanoma arising from the skin of the scrotum in a wild rabbit with testicular metastases, as an unusual metastatic site for cutaneous malignant melanoma, which has so far been reported in human patients. The growing popularity of rabbits as pet allows for a greater ability to accumulate data on the spontaneous incidence of tumours in these animals that are allowed to live natural life spans. In addition, descriptions of the biological aspects of naturally occurring tumours in the rabbit as an animal model may serve to improve current knowledge in other animal species and humans in which the same neoplasm is reported.

## Figures and Tables

**Table 1:** Haematologic and serum biochemical parameters recorded in the rabbit and physiological reference ranges [12].

Parameters	Values	Reference Ranges
Red Blood Cells (RBC) (×10^6^/μL)	5.9	4.5–6.9
Haematocrit (%)	40.2	31.3–43.3
Haemoglobin (Hgb) (g/dL)	12.3	11.0–14.4
Platelets (PLT) (×10^3^/μL)	244.8	134–567
White Blood Cells (WBC) (×10^3^/μL)	9.7	4.1–10.8
Heterophils (%)	61.2	20–75
Lymphocytes (%)	36.6	30–85
Eosinophils (%)	0.4	0–5
Basophils (%)	0.1	0–10
Monocytes (%)	1.6	0–10
Glucose (mg/dL)	145	109–161
Blood Urea Nitrogen (BUN) (mg/dL)	23.2	9–29
Creatinine (mg/dL)	1.3	1.0–2.2
Total Protein (g/dL)	7.1	6.1–7.7
Albumin (g/dL)	3.3	2.8–4.0
Globulin (g/dL)	3.8	2.1–3.7
Alanine Aminotransferase (ALT) (U/L)	36	14–80
Alkaline Phosphatase (ALP) (U/L)	24	10–140
Gamma-Glutamyl Transferase (GGT) (U/L)	7.7	0–7
Total Bilirubin (mg/dL)	0.1	0.1–0.5
Total Cholesterol (mg/dL)	18	6–65

## Data Availability

The data presented in this study are available in this article.
